# Epitope-Specific Mechanisms of IGF1R Inhibition by Ganitumab

**DOI:** 10.1371/journal.pone.0055135

**Published:** 2013-02-01

**Authors:** Frank J. Calzone, Elaina Cajulis, Young-Ah Chung, Mei- Mei Tsai, Petia Mitchell, John Lu, Ching Chen, Jilin Sun, Robert Radinsky, Richard Kendall, Pedro J. Beltran

**Affiliations:** 1 Oncology Research, Amgen Inc., Thousand Oaks, California, United States of America; 2 Biologic Discovery, Amgen Inc., Thousand Oaks, California, United States of America; 3 Metabolic Disorders, Amgen Inc., Thousand Oaks, California, United States of America; 4 Biologic Optimization, Amgen Inc., Thousand Oaks, California, United States of America; 5 Clinical Immunology, Amgen Inc., Thousand Oaks, California, United States of America; Kyushu University, Japan

## Abstract

**Background:**

Therapeutic antibodies targeting the IGF1R have shown diverse efficacy and safety signals in oncology clinical trials. The success of these agents as future human therapeutics depends on understanding the specific mechanisms by which these antibodies target IGF1R signaling.

**Methodology/Principal Findings:**

A panel of well-characterized assays was used to investigate the mechanisms by which ganitumab, a fully human anti-IGF1R antibody undergoing clinical testing, inhibits IGF1R activity. Epitope mapping using IGF1R subdomains localized the ganitumab binding site to the L2 domain. Binding of ganitumab inhibited the high-affinity interaction of IGF-1 and IGF-2 required to activate IGF1R in cells engineered for IGF1R hypersensitivity and in human cancer cell lines, resulting in complete blockade of ligand-induced cellular proliferation. Inhibition of IGF1R activity by ganitumab did not depend on endosomal sequestration, since efficient ligand blockade was obtained without evidence of receptor internalization and degradation. Clinically relevant concentrations of ganitumab also inhibited the activation of hybrid receptors by IGF-1 and IGF-2. Ganitumab was not an agonist of homodimeric IGF1R or hybrid receptors in MCF-7 and COLO 205 cells, but low-level IGF1R activation was detected in cells engineered for IGF1R hypersensitivity. This activation seems biologically irrelevant since ganitumab completely inhibited ligand-driven proliferation. The *in vivo* efficacy profile of ganitumab was equivalent or better than CR and FnIII-1 domain-specific antibodies, alone or in combination with irinotecan. CR domain-specific antibodies only blocked IGF-1 binding to IGF1R but were more potent than ganitumab at inducing homodimer and hybrid receptor downregulation *in vitro,* however this difference was less obvious *in vivo.* No inhibition of hybrid receptors was observed with the FnIII-1 domain antibodies, which were relatively strong homodimer and hybrid agonists.

**Conclusions/Significance:**

The safety and efficacy profile of ganitumab and other anti-IGF1R antibodies may be explained by the distinct molecular mechanisms by which they inhibit receptor signaling.

## Introduction

The type I insulin like growth factor receptor (IGF1R) is a heterotetrameric complex consisting of two disulfide-linked α-chains that bind IGF-1 and IGF-2 and two β-chains that include a transmembrane and a tyrosine kinase domain [1]. High-affinity binding of IGF-1 or IGF-2 to the IGF1R extracellular domain (ECD) induces a conformational change, which leads to autophosphorylation of intracellular tyrosine residues [2]. Four protein structural motifs in the IGF1R ECD have been shown to be involved in ligand binding and selectivity: L1, L2, CR, and FnIII-1 [1]. Activation of IGF1R leads to stimulation of the PI3K/Akt and other signaling pathways resulting in pro-survival and proliferative signals [3].

IGF1R is closely related to the insulin receptor (INSR), there being 35% to 70% identity between their ECDs, depending on the structural motif [1]. The selectivity of IGF1R for IGF-1 and of INSR for insulin governs the specificity of each receptor in regulating metabolism and growth in mammals [4], [5], [6]. IGF-2 activates both IGF1R and the mammalian INSR-A variant, effectively integrating signaling from both receptors [7]. Integration of IGF1R and INSR signaling can also occur through hybrid receptors, which are preferentially activated by IGF-1 or IGF-2 [7]. The role of IGF1R/INSR hybrid signaling in normal physiology and disease is an active area of investigation.

The data implicating IGF-1 and IGF-2 in cancer risk and tumor progression have positioned IGF1R as a prime oncolgy therapeutic target, anticipated to have activity against a number of human malignancies [8]. Multiple anti-IGF1R antibodies have entered clinical development during the last decade, and the safety and efficacy of these agents vary for reasons that are not clear [9], [10], [11]. There is, therefore, interest in understanding how epitope-dependent mechanisms can contribute to clinical activity. Here, we have compared the physical and biological mechanisms of IGF1R inhibition by ganitumab, an investigational anti-IGF1R antibody currently being evaluated in clinical trials, with a number of anti-IGF1R antibodies that target distinct IGF1R epitopes. Our results identify epitope-dependent mechanisms of receptor inhibition that may be important in distinguishing the clinical effects of ganitumab and other investigational anti-IGF1R antibodies.

## Materials and Methods

### Animals

Female CD1 nude mice (Charles River Laboratories, Wilmington, MA) or athymic nude mice (Harlan Laboratories, Inc., Placentia, CA) were housed in sterile cages, five per cage. The laboratory housing the cages met all Association for Assessment and Accreditation of Laboratory Animal Care International specifications. All experimental procedures were approved by the Institutional Animal Care and Use Committee of Amgen Inc. and performed in accordance with Amgen’s Institutional Animal Care and Use Committee and the United States Department of Agriculture rules and regulations. All efforts were made to minimize animal suffering.

### Antibodies

The anti-IGF1R antibodies evaluated in our study are shown in Table 1. The fully human anti-IGF1R L2 domain monoclonal antibodies, ganitumab, L2-A, L2-B, and L2-C, were isolated using a Target Quest™ human phage-displayed antibody library (Dyax Corp., Cambridge, MA). The fully human anti-IGF1R FnIII-1 domain antibodies, F1-A, F1-B, and F1-C, were isolated using the BioSite™ antibody platform (Biosite, San Diego, CA). Human (h)IGF1R(ECD)-mFc was the capture agent, and all antibodies that cross reacted with hINSR(ECD)-mFc were excluded. Murine anti-IGF1R monoclonal antibodies were obtained from a variety of sources: αIR3 from EMD Chemicals (Gibbstown, NJ), Mab 391 from R&D Systems (Minneapolis, MN), 1H7 and 26-3 from Santa Cruz Biotechnology (Santa Cruz, CA), and 24–57 from Thermo Scientific (Waltham, MA). The anti-INSR antibody 47-9 was obtained from R&D systems (Minneapolis, MN) and was used as a positive control in INSR binding experiments.

**Table 1:** Binding and Ligand Blocking Activity of IGF1R Antibodies.

Antibody	Binding Domain	Antibody Competitor	K*_D_* (nM)	IGF-1 IC_50_ (nM)	IGF-2 IC_50_ (nM)
Ganitumab	L2	Ganitumab	0.33	1.6±0.9 (7)	0.9±0.3 (7)
L2-A	L2	Ganitumab	0.10	2.5±1.4 (8)	2.2±1.4 (8)
L2-B	L2	Ganitumab	0.25	3.3+0.04 (2)	2.9+0.2 (2)
L2-C	L2	Ganitumab	0.30	3.7±2.5 (4)	3.1+1.5 (4)
αIR3	CR	αIR3	0.30	28±19 (27)	No Inhibition
Mab 391	CR	αIR3	0.02	2.6 (1)	No Inhibition
F1-A	FnIII-1	F1-B	0.30	4.3±2.8 (4)	2.4±1.0 (4)
F1-B	FnIII-1	F1-B	0.06	4.1±0.4 (5)	2.6±0.6 (5)
F1-C	FnIII-1	F1-B	0.05	4.8±0.9 (3)	2.9±0.6 (3)
1H7	FnIII-1	F1-B	0.40	47±23 (19)	31±16 (19)
24–57	FnIII-1	F1-B	0.03	17±10 (24)	7.4+4.9 (24)

The IGF1R binding domain recognized by each antibody was determined using avidin-fusion proteins. Antibody binding affinity (K*_D_*) was determined using hIGF1R(ECD) and the Biacore™ equilibrium method. Values for ganitumab binding affinity have been reported previously [12]. The number of independent determinations is shown in parentheses.

### Cell Lines and Reagents

The isolation of murine 32D myeloid cells (provided by Renato Baselga, PhD, Thomas Jefferson University, Philadelphia, PA) engineered to overexpress human IGF1R and IRS-1 (32D hIGF1R/IRS-1) and Balb/C 3T3 cells that overexpress human IGF1R (Balb/C 3T3 hIGF1R) has been described previously [3]. 32D hIGF1R/IRS-1 cells were maintained in DMEM supplemented with 10% fetal bovine serum (FBS), 10 ng/mL IL-3 (Amgen Inc., Thousand Oaks, CA), and 250 µg/mL neomycin. Balb/C 3T3 hIGF1R were maintained in DMEM supplemented with 10% bovine calf serum and 250 µg/mL neomycin. COLO 205 and MCF-7 cells were purchased from the American Type Culture Collection and maintained in RPMI plus 10% FBS. IGF-1, IGF-2, and insulin were obtained from Sigma (St. Louis, MO). INSR-B was overexpressed in Chinese Hamster Ovary (CHO) cells by stable transfection using a CMV-based promoter.

### Epitope Mapping

Constructs with an N-terminus chicken avidin tag and signal sequence included hIGF1R(ECD) (amino acids 1–932), L1 (30–179), CR (180–328), L2 (329–491), FnIII1 (488–607), FnIII2a-ID-FnIIIb (608–826), FnIII3 (827–1034), and L1-cys-rich-L2-FnIII-1-ID (1–735). Constructs with a C-terminus avidin tag were L1-cys-rich-L2 (1–491) and L1-cys-rich (1–328). IGF1R sequences were inserted into a pCEP4 vector (Invitrogen, Carlsbad, CA) containing avidin residues (1–152) for the N-terminal construct and residues (25–152) for the C-terminal construct. Expression was accomplished by transient transfection in 293 EBNA cells.

Fusion proteins (50–100 µg per 3.5×10^5^ biotin beads) were mixed with 1 µg anti-IGF1R domain-specific monoclonal antibody in 1 mL of PBS/0.5% BSA and incubated for 1 hour at room temperature. The antibody/fusion protein/biotin bead complexes were then incubated with 0.5 µg/mL FITC-labeled mouse F(ab’)2 anti-human or anti mouse secondary antibodies (Jackson ImmunoResearch Laboratories Inc., West Grove, PA), FITC-labeled anti-avidin antibody, and 0.5 µg/mL PE-labeled goat anti-human or anti-mouse secondary in 1 mL BPBS for 1 to 2 hours. FITC and/or PE were detected with a FACScan (Beckton, Dickson and Company, Franklin Lakes, NJ).

For competition experiments, biotin beads bound to fusion proteins containing the complete ECD of IGF1R were preincubated with 10–50 µg/mL unlabeled competitor antibody followed by incubation with FITC-labeled domain-specific antibody.

### Ligand Binding and Antibody Affinity

hIGF1R(ECD)-mFc contained amino acid residues 31–935 of hIGF1R fused to a mouse IgG1 Fc domain. The hINSR(ECD)-mFc contained amino acid residues 28–956 of hINSR. The fusion proteins were expressed in CHO cells and purified by protein-A sepharose chromatography. Binding reactions for antibody competition assays (in duplicate) contained 50 ng hIGF1R(ECD)-mFc preloaded on 1×10^6^ Dynal M450 paramagnetic beads coated with sheep anti-mouse IgG and ∼0.25 nM ruthenium (Ru)-labeled IGF-1 or IGF-2 (Sigma, St Louis, MO; 2 Ru per ligand molecule) in 100 µL of PBS, 0.05% Tween 20 (Mallinckrodt, St Louis, MO), 0.1% BSA, 0.01% sodium azide, and 10 pM to 1.0 µM anti-IGF1R antibody. After incubation with antibody for 2 hours at room temperature, the bound ligand was captured and detected using an IGEN™ instrument (IGEN, Gaithersburg, MD).

The effects of each anti-IGF1R antibody on ligand binding were further investigated by generating binding curves with increasing concentrations of Ru-IGF-1 or Ru-IGF-2 in the presence of excess (1 µM) antibody. Each binding assay used 75 ng hIGF1R(ECD)-mFc preloaded on anti-mouse IgG-coated MA 6000 96-well plates (Mesoscale Discovery, Gaithersburg, MD) as recommended by the manufacturer. An MSD6000 analyzer (Mesoscale Discovery) was used to detect bound Ru-labeled ligand.

The Biocore equilibrium method was used to determine antibody binding affinity as reported previously [12].

### Proliferation Assays

Serum-starved (24 hours) Balb/C 3T3 hIGF1R or 32D hIGF1R/IRS-1 cells (in triplicate), were pretreated with a range of ganitumab or control hIgG1 concentrations (1 pM to 1 µM) for 1 hour, and incubated in the presence or absence of human IGF-1 (2 nM) or IGF-2 (8 nM) for 30 minutes prior to the addition of ^3^H-thymidine (1 µCi; Perkin Elmer, Waltham, MA). Incorporation of ^3^H-thymidine was measured 24 hours later. To obtain growth curves, COLO 205 and MCF-7 cells were cultured in fresh RPMI with 10% FBS in a 96-well format (5 replicates) in the presence of control hIgG1 or anti-IGF1R antibody added at the time of cell seeding. The increase in cell confluence was monitored with an IncuCyte™ instrument (Essen Instruments, Lancaster, PA).

### IGF1R and INSR Phosphorylation

A rapid challenge assay, whereby ligand and antibodies were added simultaneously to cells for 5 minutes, was used to assess the phosphorylation status of IGF1R and INSR. COLO 205, MCF-7, and Balb/C 3T3 hIGF1R cells were starved overnight before antibody treatments in the presence of either 2 nM IGF-1 or 8 nM IGF-2. 32D hIGF1R/IRS-1 cells were starved in DMEM supplemented with 10 ng/mL IL-3 before antibody treatment with either 4 nM IGF-1 or 16 nM IGF-2. Human IGF1R was immunoprecipitated from cell lysates by incubating with 4.5 µg antibody F1-B for 2 hours, and immune complexes were captured with Protein-G agarose beads (Thermo Scientific, Rockford, MD). IGF1R was separated by 10% SDS PAGE and electroblotted onto PDVF membranes. Total and phosphorylated IGF1R were detected with the C-20 (Santa Cruz Biotechnology Inc., Santa Cruz, CA) and pY1158 (Life Technologies Inc, Grand Island, NY) antibodies, respectively, followed by an anti-goat HRP conjugate.

Mesoscale™ multiplex assays were used to quantify total and phosphorylated IGF1R, INSR, and Akt as described by the manufacturer. Analysis of IGF1R and INSR from cells treated with CR domain antibodies was performed using IgG1 high-bind plates (Mesoscale Discovery); FnIII-B was used as the capture antibody in insulin multiplex assay plates. It should be noted that hybrid IGF1R/INSR phosphorylation signals were recovered in both the INSR and IGF1R channels in the MSD IGF1R multiplex assay.

### IGF1R and INSR Levels and Receptor Internalization

Cell-surface receptor levels were determined by quantitative flow cytometry as described previously [12]. Total IGF1R or INSR levels were analyzed by western blotting using the C-20 and C-19 antibodies (Santa Cruz Biotechnology), respectively. An anti-β-tubulin antibody, D-10 (Santa Cruz Biotechnology), was included as a loading control. For *in vivo* studies, tumors were snap frozen in liquid nitrogen, homogenized in RIPA buffer, and cleared by centrifugation before analysis by western blotting. Total INSR was also detected using an insulin signaling multiplex assay (Mesoscale Discovery).

### *In Vivo* Efficacy Studies

CD1 nu/nu mice or female athymic nude mice (4–6 weeks old) were used in all experiments. 32D hIGF1R/IRS-1 cells were selected for *in vivo* growth by passaging the cells subcutaneously twice. Mice were injected subcutaneously with 5×10^6^ cells (32D IGF1R/IRS-1 or COLO 205) in Matrigel. Animals with tumors of approximately 200 mm^3^ were randomly assigned to treatment groups (10 per/group). The animals were treated intraperitoneally (IP) with anti-IGF1R antibodies or control antibodies at the indicated doses, twice weekly for the duration of the experiment. Tumor volumes and body weights were monitored twice weekly using calipers and an analytical scale, respectively. For the analysis of receptor levels and downstream signaling, the tumors were snap frozen in liquid nitrogen and processed as described previously [12].

### Statistical Analysis

Data obtained from the ligand-binding and cell-based assays were analyzed with GraphPad (Prizm™, La Jolla, CA). Differences in tumor volume were analyzed with repeated measures analysis of variance (RMANOVA) and Scheffe’s Post Hoc analysis using StatView (5.0.1, SAS Institute Inc., Cary, NC).

## Results

### Epitope Specificity

Avidin-tagged IGF1R subdomains and antibody competition assays were used to identify the structural motif recognized by the fully human monoclonal antibodies ganitumab, L2-A, L2-B, L2-C, F1-A, F1-B, and F1-C. The commercially available murine monoclonal antibodies, αIR3, Mab 391, 1H7, and 24–57, served as reference agents. The ganitumab and L2-(A–C) epitopes were localized to the L2 domain, the F1-(A–C) epitope was localized to the FnIII-1 domain, αIR3 and Mab 391 bound within the CR domain, and 1H7 and 24–57 bound within the FnIII-1 domain (Table 1; Fig. S1). Competition studies using microbeads loaded with hIGF1R(ECD)-mFc and challenged with FITC-labeled αIR3, 1H7, and ganitumab demonstrated that ganitumab does not bind to the αIR3 CR epitope or the 1H7 FnIII-1 epitope (data not shown).

### Binding Affinity

Antibody binding affinities were determined with purified hIGF1R(ECD)-mFc using the Biacore™ equilibrium method. The ganitumab disassociation constant (K*_D_* ) for hIGF1R(ECD)-mFc is 0.33 nM [12]. L2-B, L2-C, αIR3, F1-A, and 1H7 had K*_D_* between 0.25 to 0.4 nM. The other antibodies had K*_D_* between 0.02 to 0.10 nM (Table 1).

### Inhibition of Ligand Binding

**Figure 1:**
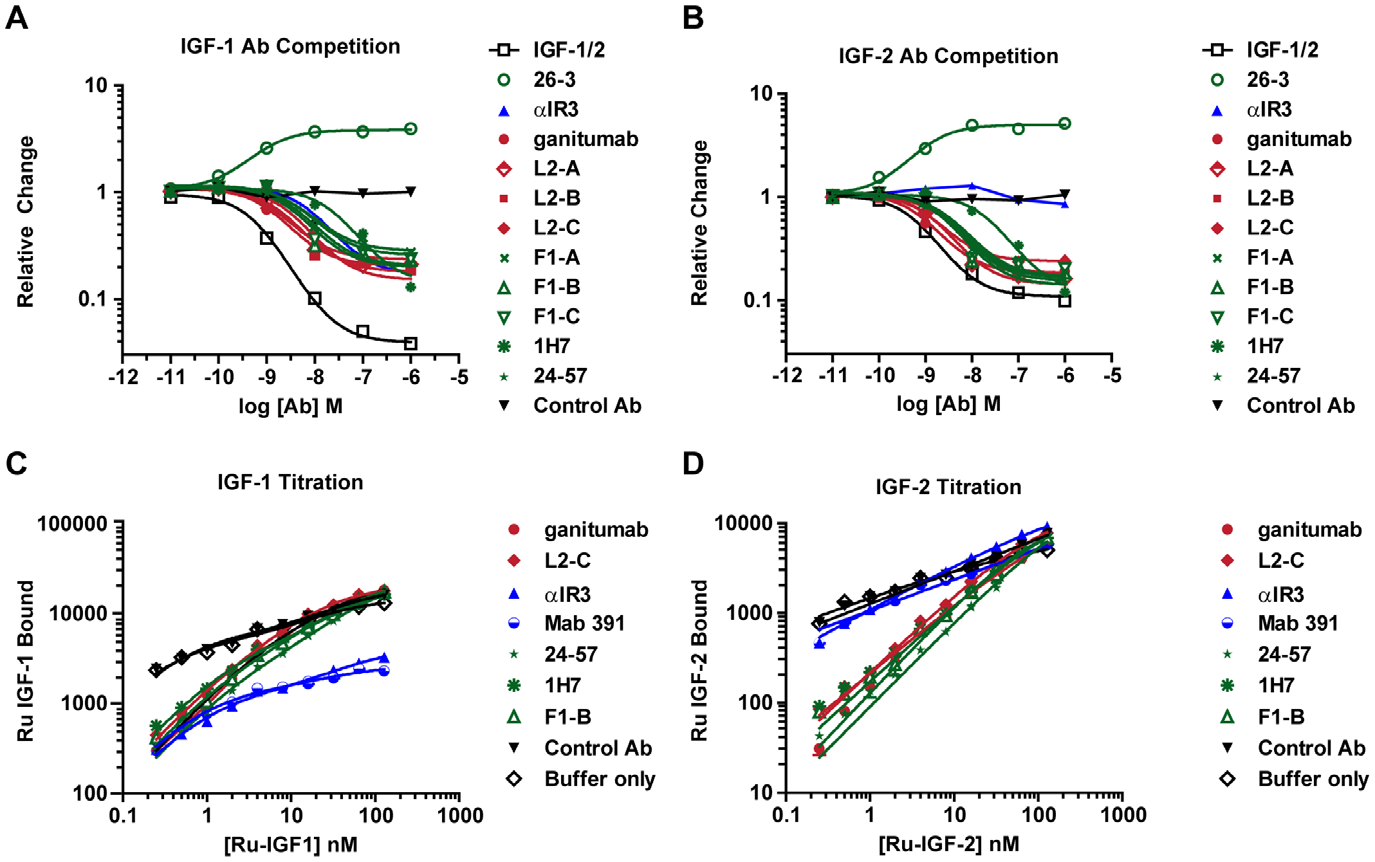
Ligand blocking activity of anti-IGF1R antibodies. Binding assays with hIGF1R(ECD)-mFc and Ru-labeled IGF-1 (A, C) and IGF-2 (B, D) were used to characterize the ligand blocking by ganitumab and other antibodies as indicated. (A, B). Antibody and ligand competition assays. Binding curves were generated by incubating hIGF1R(ECD)-mFc, Ru-labeled ligands (IGF-1/2) and increasing concentrations of antibodies or unlabeled IGF-1/2 (positive control). (C, D). The effect of excess antibody on binding of Ru-labeled ligand to hIGF1R(ECD)-mFc. Binding curves were generated with increasing concentrations of Ru-labeled IGF-1 and IGF-2 in the presence of 1 µM antibody. Background signal was subtracted before data analysis. Abbreviation: Ab, antibody.

The effect of each anti-IGF1R antibody on IGF-1 and IGF-2 binding to IGF1R was determined with purified hIGF1R(ECD)-mFc. A logarithmic-scale on the y-axis was used to highlight the differences in ligand binding obtained in the antibody survey. Antibody titrations against ∼0.25 nM Ru-labeled ligand showed that ganitumab, L2-(A–C), and F1-(A–C) inhibited IGF-1 and IGF-2 binding (Fig. 1A, B; Table 1). 1H7 and 24–57 also inhibited IGF-1 and IGF-2 binding, as reported previously [13], [14]. αIR3 and Mab 391 inhibited IGF-1 binding, but not IGF-2 binding; Ab 26-3 increased IGF-1 and IGF-2 binding. Antibody competition, when observed, reached a plateau of about 20% of ligand binding relative to control. The extent of competition obtained with unlabeled IGF-1 was significantly greater (∼2% of control). This difference between ligand and antibody saturation was not observed with L2 and FnIII-1 domain antibodies and IGF-2.

The effects of each anti-IGF1R antibody on ligand binding were further investigated by generating binding curves with Ru-IGF-1 or Ru-IGF-2 in the presence of excess antibody. All of the inhibitory IGF1R antibodies that we examined blocked the high-affinity binding observed between IGF1R and IGF-1 (at <1 nM). However, the low-affinity interaction of IGF-1 with IGF1R that was observed with L2 and FnIII-1 domain antibodies was at least 10-fold greater than observed with CR domain antibodies. The differences obtained in the antibody survey were more easily visualized in a double logarithmic plot of the binding data (Fig. 1C, D). The results obtained with IGF-2 were similar to IGF-1 except that CR domain antibodies did not block binding of IGF-2.

### Crossreactivity With INSR

None of the IGF1R antibodies used in our study cross reacted with purified hINSR(ECD)-mFc or prevented binding of insulin to hINSR(ECD)-mFc (data not shown). Activation of INSR by IGF-1, IGF-2, and insulin was not inhibited by up to 1 µM ganitumab, Mab 391, or F1-B (Fig. S2).

### Activity and eExpression of INSR and IGF1R in Cell Lines Used for Antibody Comparisons

The levels of IGF1R and INSR and the effects of IGF-1, IGF-2, and INS on IGF1R and INSR activation for each cell line are summarized in Table S1. Across cell lines, activation of IGF1R by IGF-1 occurred within a narrow range of concentrations (EC_50_∶ 0.2 to 2.7 nM). A similar pattern was observed with IGF-2, but the concentration range was shifted 10-fold (EC_50_∶2.1 to 33 nM). The effects of IGF-1 and IGF-2 on INSR were only assessed in the COLO 205 and MCF-7 cell lines. For INSR, the EC_50_ for IGF-1 was 20-fold lower than the EC_50_ for IGF-2. The EC_50_ for INS in COLO 205 and MCF-7 cells was 2 and 3.5 times higher than the EC_50_ for INS in CHO cells engineered to overexpress hINSR.

### Inhibition of Cell Proliferation

**Figure 2:**
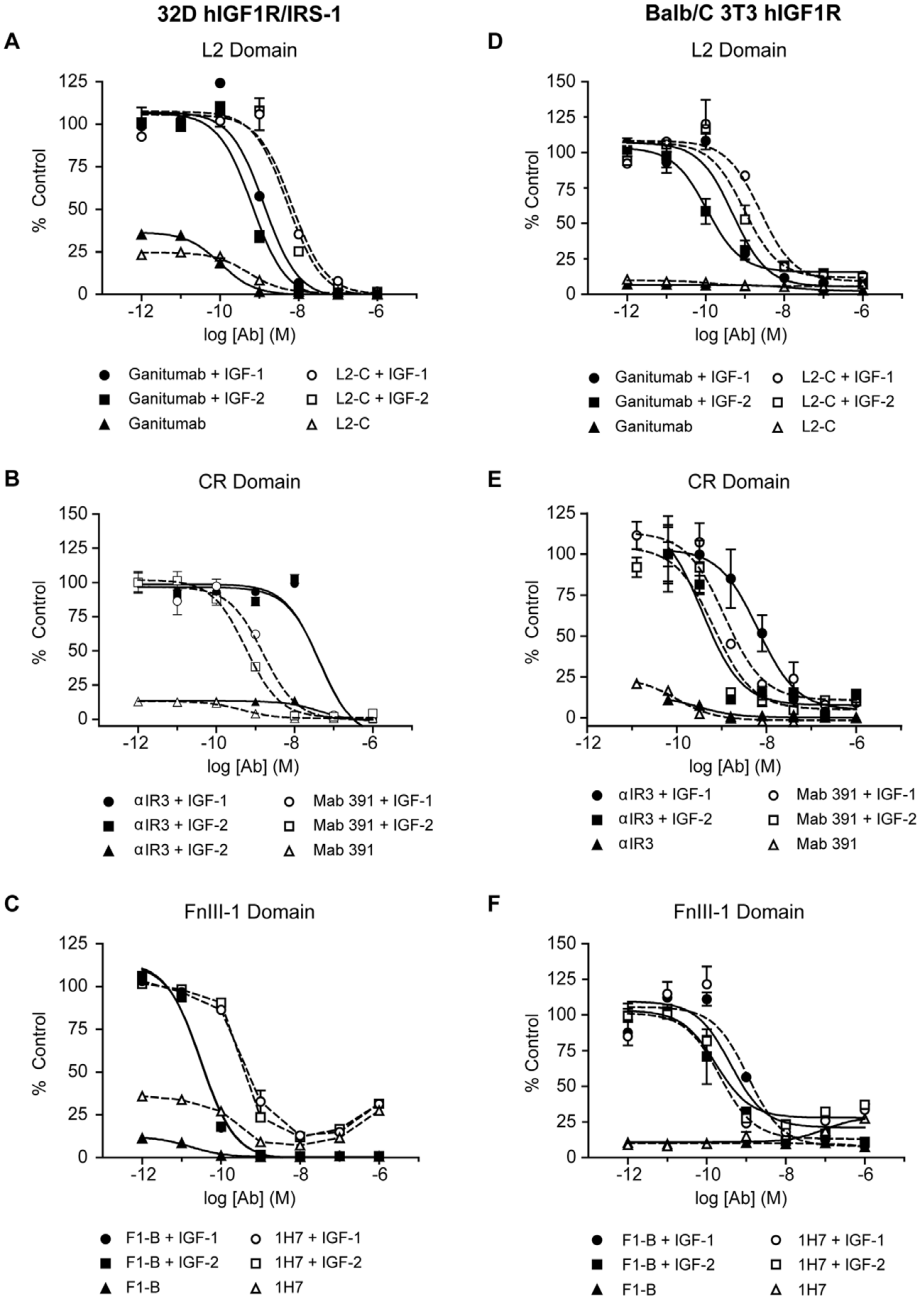
Antibody inhibition of ^3^H-thymidine incorporation in engineered cell lines. 32D hIGF1R/IRS-1 cells (A–C) and Balb/C 3T3 hIGF1R cells (D–F) were treated with increasing concentrations of the indicated domain-specific antibodies in the presence or absence of 2 nM IGF-1 or 8 nM IGF-2. (A–C). 32D hIGF1R/IRS-1 cells treated for 48 hours with antibody and growth factors in 5% FBS, RPMI. ^3^H-thymidine labeling occurred between 24–48 hours. (D–F). Balb/C 3T3 hIGF1R cells were serum-starved in DMEM +0.1% BSA overnight before treatment with antibody and growth factor for an additional 24 hours in the presence of ^3^H-thymidine. A human anti-CD20 control antibody had no significant effect on ^3^H-thymidine incorporation (data not shown).

The *in vitro* effects of anti-IGF1R antibodies on cell proliferation are summarized in Table 2. Ganitumab completely inhibited IGF-1- and IGF-2-stimulated ^3^H-thymidine incorporation in 32D hIGF1R/IRS-1 and Balb/C 3T3 hIGF1R cells (Fig. 2A, D). Ganitumab alone reduced ^3^H-thymidine incorporation below baseline in the 32D hIGF1R/IRS-1 cells indicating that the antibody is effective against the IGF-1 and IGF-2 present in the FBS-containing assay medium (Fig. 2A). No evidence of receptor activation/stimulation was observed in cells treated with ganitumab alone (Fig. 2A, D). The IC_50_ obtained with L2-(A–C), αIR3, Mab 391, F1-(A–C), and 1H7 varied by 3 orders of magnitude (Table 2). Partial stimulation of ^3^H-thymidine incorporation into 32D hIGF1R/IRS-1 cells was observed with antibody 1H7, but this agonistic activity was not observed with Balb/C 3T3 hIGF1R cells (Fig. 2C, F).

**Table 2:** Effects of Anti-IGF1R Antibodies on Cell Proliferation.

Antibody	32D hIGF1R/IRS-1			Balb/C 3T3 hIGF1R		
	IGF-1 IC_50_ (nM)	IGF-2 IC_50_ (nM)	Agonistic Activity	IGF-1 IC_50_ (nM)	IGF-2 IC_50_ (nM)	Agonistic Activity
Ganitumab	1.1±0.7 (3)	0.6±0.4 (3)	0.01±0.01 (3)	1.5±1.1 (3)	0.8±0.7 (3)	0.44±0.1 (3)
L2-A	1.8±1.6 (4)	1.2±1.1 (4)	0.03±0.03 (4)	1.6±1.4 (3)	1.2±1.7 (3)	1.0±0.2 (3)
L2-B	2.6	1.7	0.15	2.9	0.8	1.1
L2-C	6.1	5.3	0.02	2.5	1.0	0.7
αIR3	41	42	0.0	ND	ND	ND
Mab 391	1.4±0.2 (3)	0.8±0.2 (3)	0.0 (3)	1.3	2.9	1.8
F1-A	0.03	0.02	0.1	0.2	0.2	2.5
F1-B	0.03	0.03	0.05	0.4, 0.7 (2)	0.4, 0.6 (2)	2.0, 3.1 (2)
F1-C	0.03, 0.06 (2)	0.10, 0.12 (2)	0.01, 0.44 (2)	0.3	0.3	1.0
1H7	0.6±0.2 (3)	0.2±0.1 (3)	0.9±0.1 (3)	1.0±0.1 (3)	0.2±0.07 (3)	0.6±0.3 (3)
24–57	0.1, 1.2 (2)	0.05, 0.2 (2)	0.14, 0.49 (2)	0.9	0.08	0.7

The IC_50_ for ligand inhibition of cell proliferation was derived from the antibody dose-titrations curves. The number of replicate experiments is shown parenthesis. ND, not determined.

Ganitumab and Mab 391 treatment inhibited the proliferation of COLO 205 and MCF-7 cells in 10% FBS growth media 1.2- to 1.8-fold. In contrast, F1-B increased the growth of COLO 205 cells and had no effect on the growth of MCF-7 cells (Fig. S3).

### Internalization and Degradation of IGF1R and INSR

**Figure 3:**
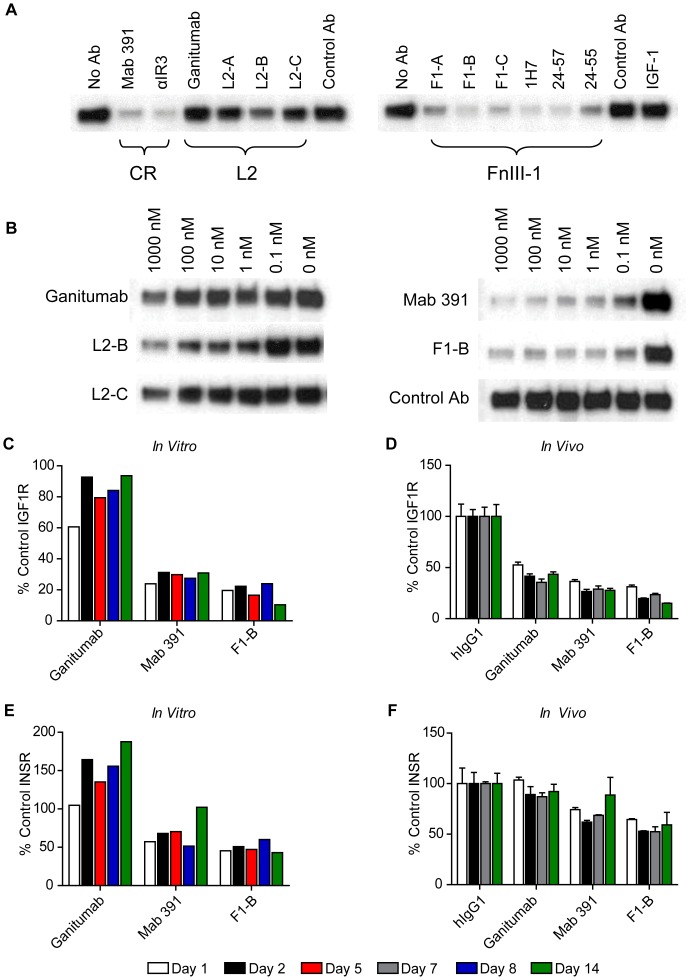
Analysis of antibody-mediated receptor internalization and degradation. Levels of IGF1R and INSR-B subunit were determined by direct western and immunoblot quantitation after treatment of COLO 205 cells or tumors with antibodies as indicated. An equal amount (20 µg) of total protein extract was loaded in each lane. (A). COLO 205 cells in 10% FBS growth medium were treated with 250 nM anti-IGF1R antibody, control anti-CD20 antibody, or IGF-1 (100 nM) for 17 hours, and IGF1R levels were determined. (B). COLO 205 cultures were treated for 17 hours with increasing concentrations (0 to 1000 nM) of representative L2, CR, and FnIII-1 domain antibodies and IGF1R levels were determined. (C). COLO 205 cells were grown in the presence of 250 nM antibody over a 2-week period to determine the long-term effects of ganitumab, Mab 391, and F1-B on IGF1R expression. The antibody was replenished when the cells were subcultured. All signals were normalized to the IGF1R signal obtained with the control antibody at each time point. (D). Mice with established (200–300 mm^3^) subcutaneous tumors were treated with ganitumab, Mab 391, or F1-B (300 µg/dose; IP). At the indicated time points, 3 animals were sacrificed and IGF1R levels were determined. % control is the signal obtained for an individual animal divided by the mean for the control antibody multiplied by 100 for each treatment group. Total INSR level was determined in the same cell (E) and tumor (F) extracts used for the long-term analysis of IGF1R (C, D).

Treatment with CR and FnIII-1 domain antibodies was consistently associated with a ≥70% reduction in total IGF1R (Fig. 3A). Internalization occurred within 15 minutes of antibody application as determined by confocal microscopy with FITC-labeled antibodies (data not shown), and receptor degradation was detected after 4 hours of treatment (data not shown). The dose dependence of IGF1R downregulation was examined for selected antibodies in COLO 205 cells (Fig. 3B). Mab 391 and F1-B exhibited the highest potency (IC_50_<0.1 nM) followed by L2-B (IC_50_ 0.1–1 nM). Ganitumab and L2-C downregulation of IGF1R only occurred at antibody concentrations >100 nM (Fig. 3B). Extending ganitumab treatment to 14 days did not reduce IGF1R levels, rather levels tended to increase (Fig. 3C). Similar data were obtained in the MCF-7 cell line (Fig. S4A). The ability of ganitumab, Mab 391, and F1-B to downregulate IGF1R expression was also evaluated in mice bearing established COLO 205 tumors treated twice weekly with 300 µg antibody for up to 14 days (Fig. 3D). *In vivo*, ganitumab, Mab 391, and F1-B reduced total IGF1R by 50% to 60%. Similar results were obtained with MCF-7 cells, which express a higher ratio of IGF1R to INSR than COLO 205 cells (Fig. S4B).

The ability of ganitumab, Mab 391, and F1-B to downregulate INSR was also evaluated *in vitro* and *in vivo*. Total INSR increased slightly in COLO 205 cells exposed to ganitumab (Fig. 3E). In contrast, Mab 391 and F1-B reduced total INSR levels 40% to 50% in COLO 205 cells (Fig. 3E) and 15% to 25% in MCF-7 cells (Fig. S4C) with maximum downregulation after one day of antibody incubation. Ganitumab did not significantly alter total INSR levels in COLO 205 tumors, whereas Mab 391 and F1-B reduced INSR by 20% to 30% (Fig. 3F). Similar effects of ganitumab, Mab 391, and F1-B were observed in MCF-7 xenografts (Fig. S4D **)**.

### IGF1R and INSR Activation

**Figure 4:**
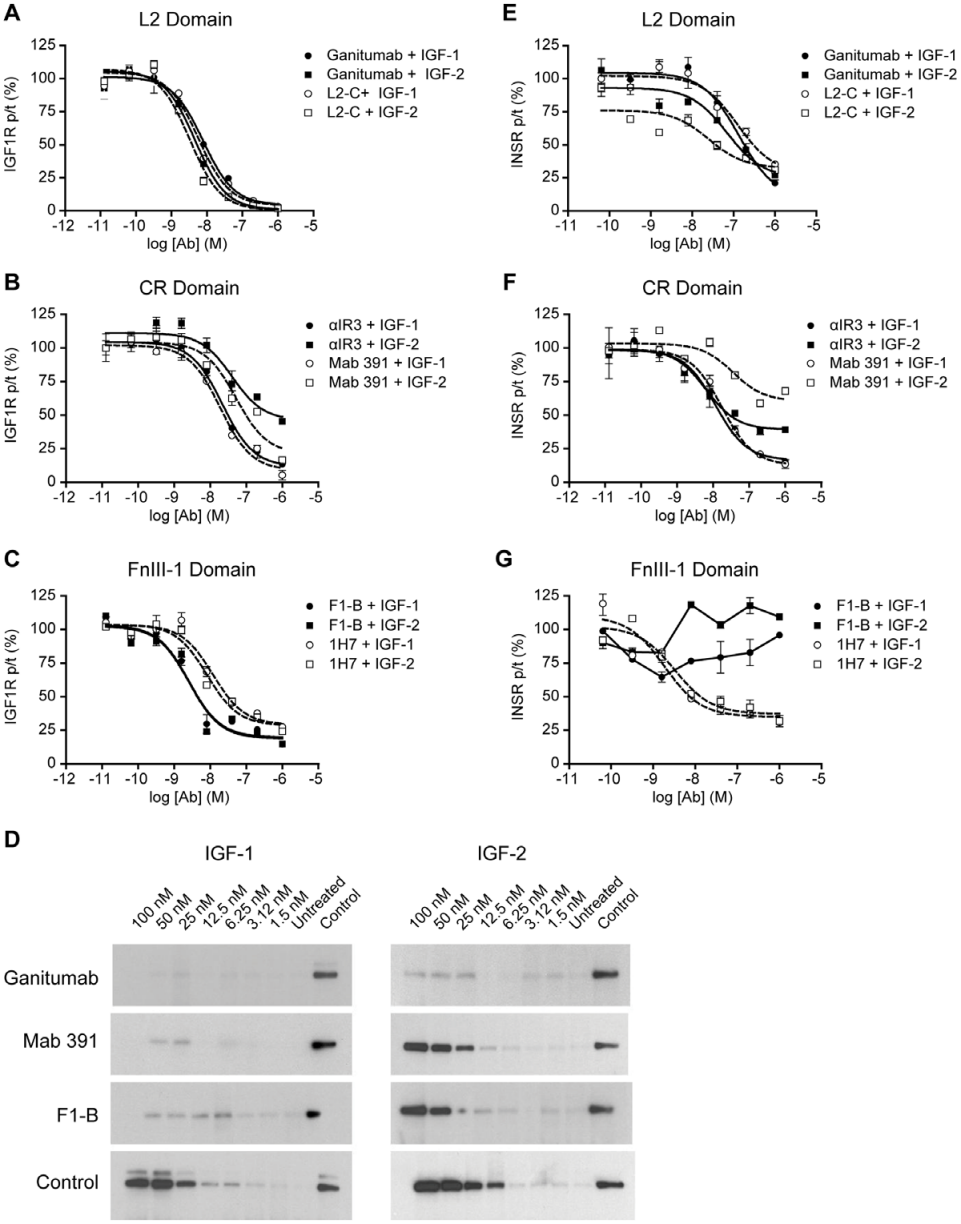
Antibody effects on IGF1R and INSR activation by IGF-1 and IGF-2 in COLO 205 cells. (A–C). Determination of antibody IC_50_ for IGF1R inhibition. Serum-starved COLO 205 cells were treated for 20 minutes simultaneously with either 2 nM IGF-1 or 8 nM IGF-2 and antibody as indicated. Total (t) and tyrosine autophosphorylation (p) IGF1R after αIR3 and Mab 391 treatment were determined in duplicate using an MSD assay with F1-B as the capture agent. All other receptor measurements were obtained using an MSD multiplex in which IGF1R and INSR were simultaneously determined. Titration with a non-specific anti-CD20 antibody increased receptor autophosphorylation to maximum of about 110% of control (data not shown). (D). Titrations with IGF-1 and IGF-2 in the presence of excess antibody. Balb/C 3T3 hIGF1R cells were treated for 5 minutes with increasing concentrations of IGF-1 or IGF-2 in the presence of anti-IGF1R or control antibody (1 µM). Total (no change, not shown) and pIGF1R were assayed by immunoprecipitation and western blotting. The control extract included in each immunoblot was prepared from cells stimulated with 100 nM IGF-1 or IGF-2 in the presence of 1 µM anti-CD20 antibody. (E–G). Determination of INSR IC_50_ for antibody inhibition. INSR tyrosine autophosphorylation was determined using the IGF1R/INSR multiplex assay with the same COLO 205 cell extracts used for IGF1R.

To distinguish antibody effects on IGF1R homodimer and hybrid receptors, inhibition of ligand-induced activation of IGF1R and INSR was characterized in four cell lines that contained different levels of IGF1R and INSR (Table S1). Each anti-IGF1R antibody was an effective inhibitor of IGF-1-induced activation of IGF1R regardless of the epitope, although differences in IC_50_ were observed (Table 3). The L2 and FnIII-1 domain antibodies were more effective inhibitors of IGF-2-induced activation of IGF1R than the CR domain antibodies (Table 3; Fig. 4A–C: COLO 205; Fig. S5A–C: MCF-7).

**Table 3:** Effect of Anti-IGF1R Antibodies on IGF1R and INSR Autophosphorylation.

			IC_50_ (95% Confidence Intervals), nM					
Cell Line	Receptor	Ligand	Ganitumab	L2-C	αIR3	Mab 391	F1-B	1H7
COLO 205	IGF1R	IGF-1	7.8 (4.9–12)	5.8 (3.3–10)	22 (13–36)	19 (12–29)	2.6 (1.4–4.5)	11 (6.3–23)
		IGF-2	4.6 (2.8–7.6)	3.3 (2.1–5.3)	Partial Inhibition	Partial Inhibition	2.6 (1.2–5.8)	8.2 (4.5–15)
	INSR	IGF-1	270 (89–808)	>250	26 (14–48)	24 (14–42)	No Inhbition	2.8 (1.2–6.7)
		IGF-2	240 (102–565)	>250	13 (8–21)	14 (7–28)	No Inhbition	3.1 (1.2–8.3)
MCF-7	IGF1R	IGF-1	7.6 (4.2–14)	4.9 (2.2–11)	22 (6.3–76)	9.0 (3.3–22)	0.7 (0.5–1.0)	6.0 (2.5–15)
		IGF-2	0.9 (0.6–1.2)	0.6 (0.3–1.0)	Partial Inhibition	Partial Inhibition	2.9 (1.4–6.0)	5.9 (1.8–19)
	INSR	IGF-1	136 (57–321)	84 (22–322)	26 (14–48)	18 (11–28)	No Inhibition	4.3 (0.8–23)
		IGF-2	70 (25–190)	13 (2.4–76)	13 (8–21)	35 (9–138)	No Inhibition	6.8 (0.9–48)
Balb/C 3T3 hIGF1R	IGF1R	IGF-1	5.3 (2.5–11)	4.8 (1.8–13)	36 (10–89)	7.4 (0.4–14)	4.2 (1.4–13)	21 (6.6–69)
		IGF-2	5.1 (1.8–15)	5.8 (2.0–17)	No Inhibition	No Inhibition	4.6 (1.5–14)	35 (13–94)
32D hIGF1R/IRS-1	IGF1R	IGF-1	4.8 (1.6–14)	1.3 (0.7–2.3)	65 (2.4–172)	53 (28–102)	8.4 (1.1–61)	1.5 (0.7–3.1)
		IGF-2	0.7 (0.5–1.1)	1.7 (1.0–2.9)	No Inhibition	No Inhibition	18 (41–77)	0.9 (0.3–2.3)

The lC_50_ for antibody inhibition of ligand-induced receptor activation was extracted from antibody dose titrations. The 95% confidence intervals of the curve fit are given in parentheses. Agonistic potential is the maximum signal obtained with antibody (0.1–1 µM) in the absence of ligand divided by the signal without antibody and ligand.

To further characterize the observed inhibitory effects, a rapid ligand challenge assay was developed to minimize receptor inhibition by internalization. Balb/C 3T3 hIGF1R cells were treated with increasing IGF-1 or IGF-2 in the presence of 1 µM ganitumab, Mab 391, or F1-B, and the incubation time was reduced to 5 minutes. Under these conditions, ganitumab inhibited IGF-1- and IGF-2-induced activation of IGF1R at all ligand concentrations (Fig. 4D). Mab 391 and F1-B inhibited IGF-1-induced, but not IGF-2-induced activation of IGF1R (Fig. 4D).

Ganitumab and L2-C inhibited IGF-1- and IGF-2-induced activation of INSR; however, the CR domain antibodies (αIR3 and Mab 391) were more potent inhibitors of IGF-1-induced activation of INSR than the L2 domain antibodies (Table 3; Fig. 4E, F: COLO 205; Fig. S5D, E: MCF-7). However, as observed with IGF1R, only partial inhibition of IGF-2-induced activation of INSR was achieved with αIR3 and Mab 391. Surprisingly, F1-B did not inhibit IGF-1- or IGF-2-induced activation of INSR (Table 3; Fig. 4G: COLO 205; Fig. S5F: MCF-7).

### Agonistic Potential of Anti-IGF1R Domain Antibodies

**Figure 5:**
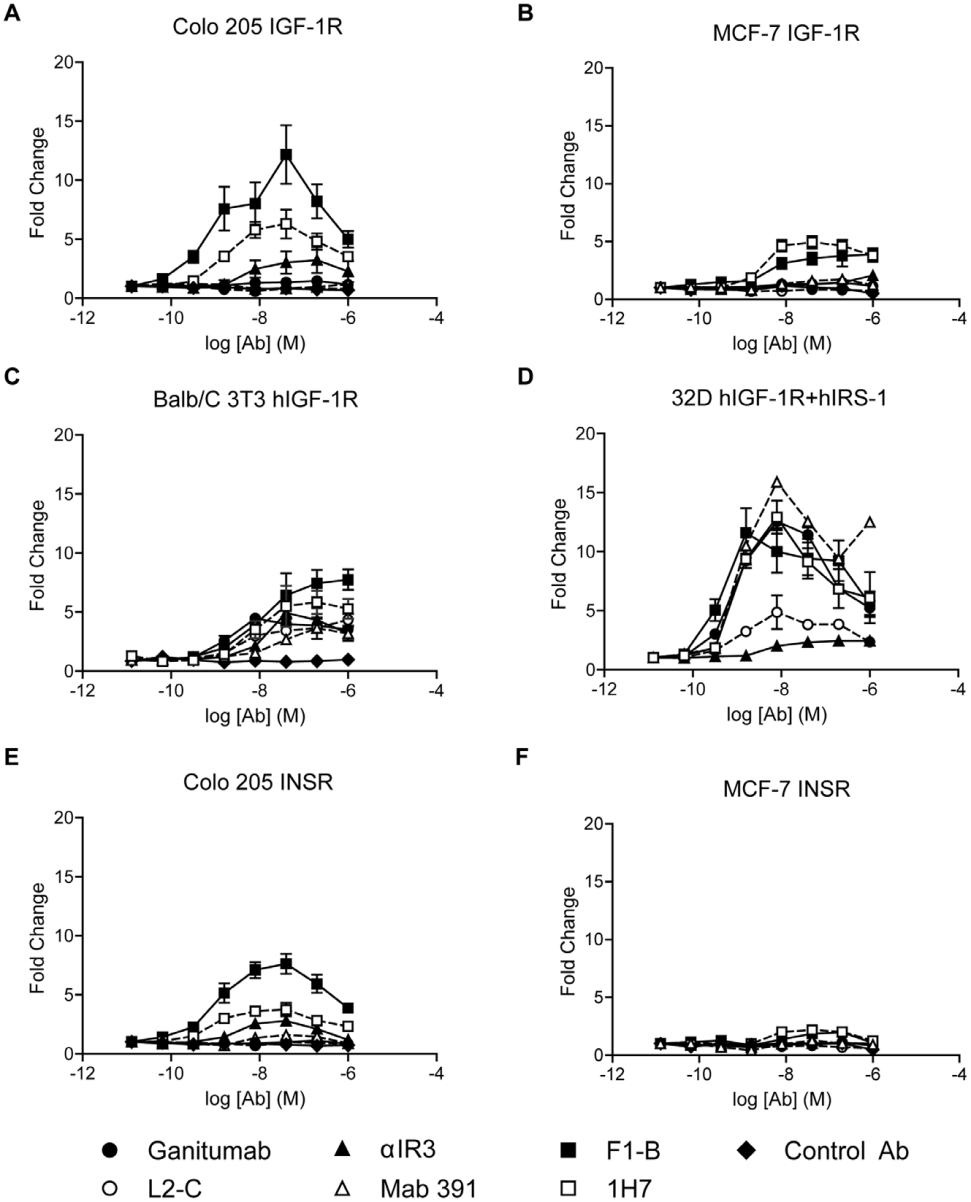
Analysis of agonistic activity of anti-IGF1R antibodies. Serum-starved cells were treated with antibody for 20 minutes without the addition of IGF-1 or IGF-2 under conditions identical to those used for the analysis of receptor inhibition. Total and phosphorylated (p) IGF1R and INSR were determined; the fold change relative to background is plotted against antibody dose. (A–D). Determination of IGF1R tyrosine phosphorylation with COLO 205, MCF-7, Balb/C 3T3 hIGF1R, and 32D hIGF1R/IRS-1 cells. E,F. Analysis of INSR phosphorylation in response to antibody treatment in COLO 205 and MCF-7 cells. Partial IGF1R agonism was also observed for F1-A, F1-C, 24-57, and L2-B in a qualitative survey with Balb/C 3T3 hIGF1R cells (data not shown).

The agonistic potential of anti-IGF1R domain antibodies against IGF1R, INSR, and hybrid receptors in the absence of ligands was investigated (Fig. 5). The FnIII-1 domain antibodies (F1-B, 1H7) exhibited a consistently high level of IGF1R agonism (4- to12-fold times over baseline) in each cell line examined (Fig. 5A–D). Ganitumab and L2-C did not significantly stimulate IGF1R phosphorylation in COLO 205 and MCF-7 cells (Fig. 5A, B). However, partial agonism with L2 antibodies was observed in Balb/C 3T3 hIGF1R cells (4-fold) and 32D hIGF1R/IRS-1 cells (4- to 12-fold) (Fig. 5C–D). IGF1R stimulation (3-fold) was obtained with αIR3 in COLO 205 cells (Fig. 5A), and both of the CR domain antibodies exhibited partial agonism in 32D hIGF1R/IRS-1 cells (Fig. 5D). In general, IGF1R agonism was maximal at an antibody concentration of approximately 10 nM.

Ganitumab, L2-C, and Mab 391 did not increase INSR autophosphorylation, but αIR3, F1-B, and 1H7 stimulated INSR in the absence of ligand (Fig. 5E, F).

### Antitumor Activity of Anti-IGF1R Domain Antibodies

**Figure 6:**
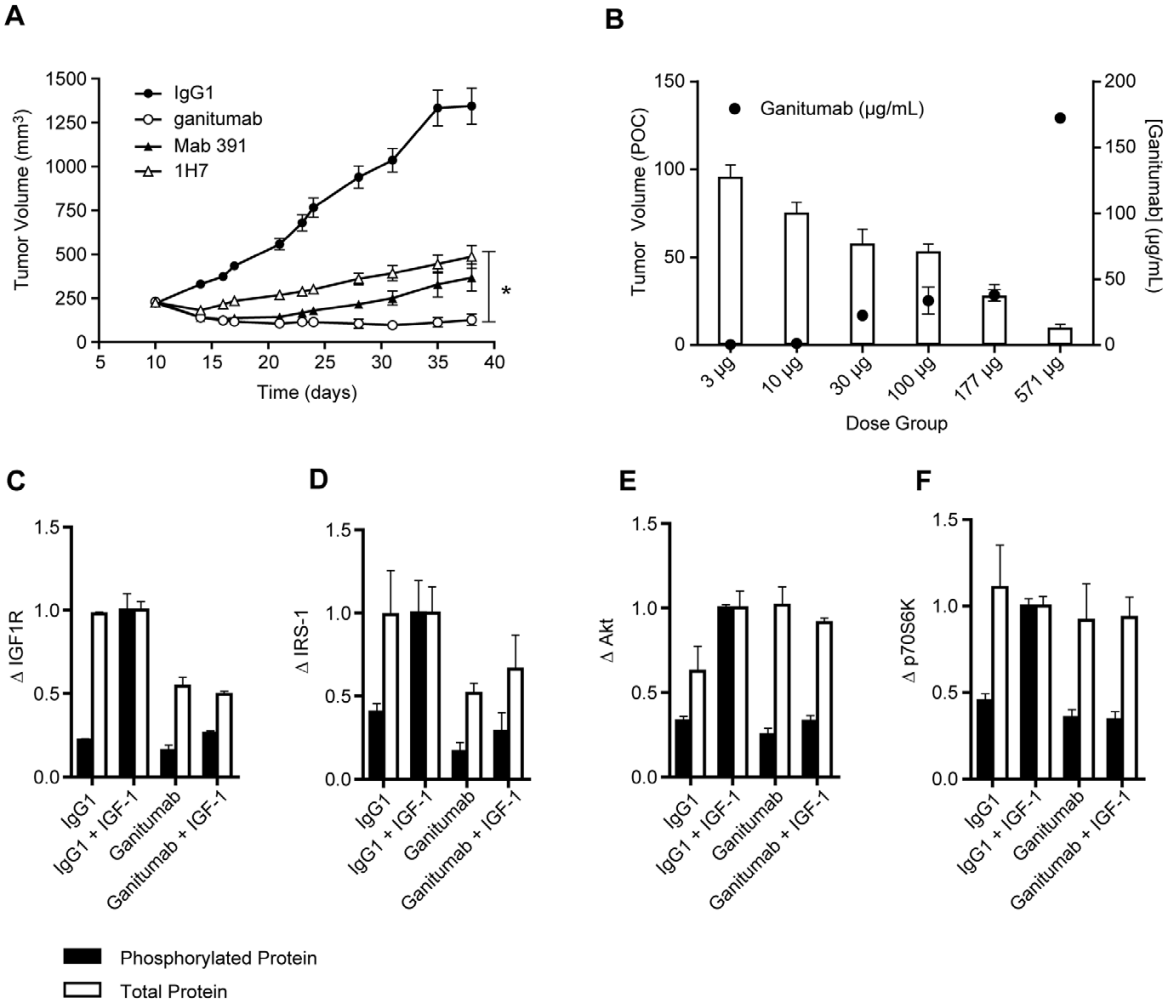
Inhibition of the growth of 32D hIGF1R/IRS-1 tumor xenografts. (A). TGI by ganitumab, Mab 391, or 1H7. Mice bearing 32D hIGF1R/IRS-1 xenografts (∼200 mm^3^) were randomly assigned into treatment groups (n = 10) and treated with antibody (300 µg, twice per week, IP) for the duration of the experiment. Tumor volumes and body weights were measured twice per week using calipers and an analytical scale, respectively. Data are presented as mean tumor volume ± standard error of the mean. (B). Relationship between ganitumab serum concentration and TGI. Ganitumab serum concentration (µg/mL) was determined 2 hours after the final dose and plotted as the mean ± standard deviation. (C–F). Inhibition of IGF1R signaling *in vivo*. Mice bearing 32D hIGF1R/IRS-1 xenografts (∼400 mm^3^) were randomly assigned into treatment groups (n = 6) and treated with ganitumab or IgG1 control for 6 hours. Three animals from each group were then challenged with 5 µg IGF-1 or PBS IV for 15 minutes. Total and phosphorylated IGF1R, IRS-1, Akt, and p70S6K in tumor extracts (100 µg) were determined with MSD multiplex assays. No significant changes in GSK3β were observed (data not shown). *p<.0003.

The antitumor activity of the domain-specific antibodies was determined using 32D hIGF1R/IRS-1 subcutaneous xenografts (Table 4). Potent tumor growth inhibition (TGI) ranging from 75% TGI to tumor regression was achieved with L2, CR, and FnIII-1 domain antibodies in this model. Ganitumab and L2-C treatment led to tumor regressions (43% and 52%) at the maximum dose tested (300 µg, IP, twice weekly; Table 4, Fig. 6A). Using a range of ganitumab doses (3 to 300 µg), the *in vivo* ED_50_ for 32D hIGF1R/IRS-1 TGI was determined to be 1.5 µg/mL (Fig. 6B). Total or almost total TGI was achieved with the CR-domain antibodies Mab 391 and αIR3 and the FnIII-1 domain antibodies F1-A and F1-B (Table 4, Fig. 6A).

**Table 4:** Effects of Anti-IGF1R Antibodies on Tumor Growth Inhibition of 32D higf1r/IRS-1 Xenografts.

Antibody	% TGI (100 µg)*	Plasma Concentration (µg/mL)	% TGI (300 µg)*	Plasma Concentration (µg/mL)
Ganitumab	87	45	43% regression	285
L2-A	81	58	91	143
L2-B	76	100	84	472
L2-C	98	172	52% regression	324
αIR3	95	NA	99	NA
Mab 391	81±8.5	487	92±6.6	540
F1-A	96	25	100	208
F1-B	8% regression	133	100	301
F1-C	80	102	85	361
1H7	61	NA	76	NA

Anti-IGF1R antibodies were administered IP twice per week at 100 or 300 µg/dose for the duration of the experiment. TGI was calculated on the last day of each study and expressed relative to the initial and final mean tumor volume of the control group. The significance of TGI against the control group was calculated using RMANOVA followed by a post-hoc Scheffe’s test. Serum samples were collected 2 hours after the last antibody dose (n = 3). TGI for Mab 391 is the mean and standard deviation of 4 xenograft experiments. *All TGI values were significant against the control IgG1 group, p<0.05. NA: not available.

To determine the effect of ganitumab on IGF1R pathway signaling *in vivo*, established 32D hIGF1R/IRS-1 tumors that were pretreated for 6 hours with ganitumab or control antibody were challenged with IGF-1 for 15 minutes. Pretreatment with ganitumab led to almost complete inhibition of IGF-1-induced activation of IGF1R, IRS-1, Akt, and p70S6K (Fig. 6C–F). Ganitumab treatment without IGF-1 stimulation did not show any evidence of IGF1R agonism (Fig. 6C). Mechanism-of-action studies on ganitumab-treated, established 32D hIGF1R/IRS-1 tumors showed complete inhibition of Ki67 labeling (data not shown). This observation is consistent with the complete inhibition of ^3^H-thymidine incorporation demonstrated *in vitro* (Fig. 2A).

### Antitumor Activity of Anti-IGF1R Domain Antibodies in Combination with Irinotecan

**Figure 7:**
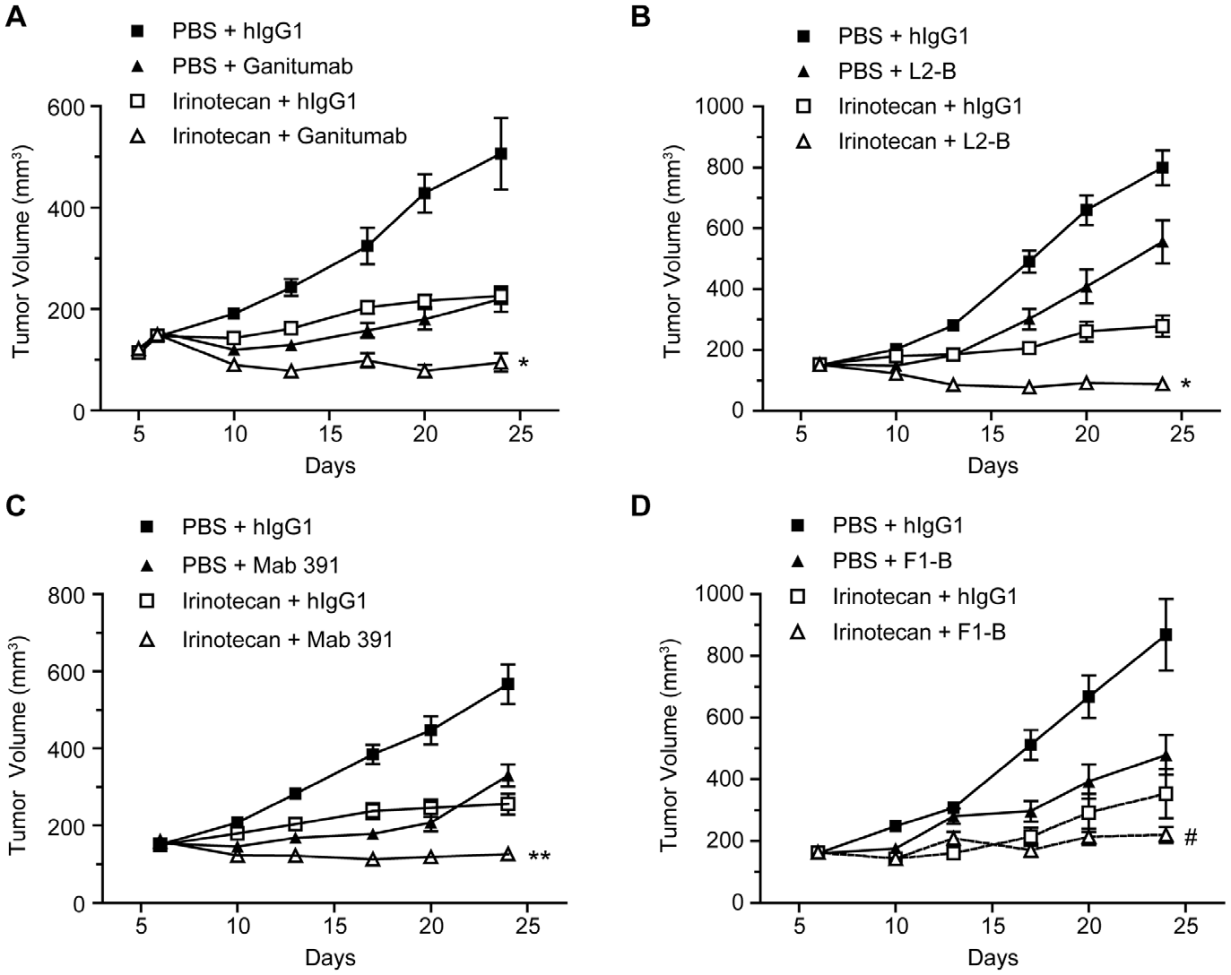
Effects of anti-IGF1R antibodies in combination with irinotecan in the COLO 205 xenograft model. Mice bearing COLO 205 xenografts (∼200 mm^3^) were randomly assigned into treatment groups (n = 10) and treated with antibody (300 µg, twice per week, IP), irinotecan (35 mg/kg, once per week, IV), or the combination of both agents for the duration of the experiment. Tumor volumes and body weights were measured twice per week using calipers and an analytical scale, respectively. Data are presented as mean tumor volume ± standard error of the mean. The combination of anti-IGF1R antibodies and irinotecan: (A). ganitumab; (B). L2-B; (C). Mab 391; and (D). F1-B. *p<.0003 versus either single agent; **p<.001 versus either single agent; ^#^ *p*>0.004 versus F1-B alone and *p* = 0.55 versus irinotecan alone.

COLO 205 xenografts were used to evaluate the effect of combining domain-specific IGF1R antibodies with irinotecan. Ganitumab (300 µg, IP, twice weekly) and irinotecan (35 mg/kg, IV, once per week) treatment significantly inhibited tumor growth by approximately 75% as single agents. Statistically significantly better TGI (p<0.0003 vs either agent) resulting in regressions was achieved by the combination of ganitumab and irinotecan (Fig. 7A). Similar results were observed with L2-B (Fig. 7B) and Mab 391 (Fig. 7C). The addition of F1-B to irinotecan did not lead to statistically significantly better efficacy than irinotecan alone (Fig. 7D); similar results were obtained with the other FnIII-1 domain antibodies in combination with irinotecan (data not shown). The level of total IGF1R in COLO 205 tumors harvested at the end of treatment was consistently reduced (≥70%) regardless of the IGF1R domain targeted (data not shown).

## Discussion

Safety and efficacy data emerging from phase II clinical studies have highlighted the need to better elucidate the individual characteristics of the multiple anti-IGF1R antibodies currently in clinical development. In this study, we used a panel of well-characterized *in vitro* and *in vivo* assays to investigate the mechanisms by which ganitumab inhibits IGF1R. We observed notable differences among domain specific anti-IGF1R antibodies with regards to their ability to 1) inhibit ligand binding and ligand-induced activation of IGF1R and INSR, 2) downregulate IGF1R and INSR expression, 3) inhibit cell proliferation and tumor growth, and 4) activate IGF1R and INSR in the absence of ligand.

Previous studies have shown that IGF1R antibodies can vary significantly in their ability to block ligand binding [14], [15]. Ganitumab and other L2 domain antibodies appear to be the most efficient at inhibiting both IGF-1 and IGF-2 from binding to IGF1R. This is an important therapeutic criterion as all human cancers are exposed to IGF-1 and IGF-2 due to hepatic endocrine synthesis, tumor stroma expression, and autocrine activation [16]. In addition, IGF-2 is a well-described transforming growth factor whose elevated expression as a result of loss of imprinting can be found in a considerable percentage of human cancers [17]. Our data on inhibition of ligand binding appears to have revealed a difference in ligand binding affinity to ligand-binding sites 1 and 2 on IGF1R. We used the De Meyts model for negative cooperative ligand binding or ‘alternative bivalent crosslinking’ [2], [18] to interpret the results of our ligand-binding assays. The model defines a pair of ligand binding surfaces, site 1 and site 2, within each IGF1R α-chain that are held in an antiparallel arrangement within the receptor dimer. High-affinity ligand binding occurs when the α-chains are crosslinked by the occupation of one subsite pair (site 1 of α-chain 1 and site 2 of α-chain 2) with a bivalent IGF-1 or IGF-2 monomer. Antibody competition and ligand titration curves both indicate that ganitumab inhibits the high-affinity interaction (∼0.2 nM K*_D_*) of IGF-1 and IGF-2 associated with negative-cooperative IGF1R ligand binding [18]. However, the ligand titrations also revealed that preloading with excess (1 µM) ganitumab permits IGF-1 and IGF-2 binding at a reduced affinity site (1–100 nM) that is within the range described for negative cooperative ligand binding [2]. The binding data are consistent with the idea that ganitumab holds IGF1R in a configuration that prevents alternative bivalent crosslinking, but that is nonetheless open to the non-cooperative, low-affinity binding of multiple ligand molecules. This low-affinity binding in the ganitumab-bound state is functionally irrelevant since IGF1R activation was not detected with IGF-1 and IGF-2 (at concentrations ≤100 nM) after antibody preloading in the rapid challenge assay, designed to minimize receptor inhibition by internalization. The surfaces that remain accessible to ligand binding in the ganitumab-bound receptor remain to be determined, but it is clear that these include contacts shared by IGF-1 and IGF-2.

IGF1R/INSR hybrids have been identified in multiple human cancers. Their expression and activity may be, at least in some tumor types, even more relevant than that of IGF1R homodimers [7], [19]. Therefore, it is critical to ensure that hybrid receptor activity is also inhibited with IGF1R therapeutic antibodies at clinically meaningful concentrations. Ganitumab and the other L2 domain antibodies were effective inhibitors of IGF-1- and IGF-2-induced activation of hybrid receptors, albeit at a greater IC_50_ compared with IGF1R homodimers (∼50-fold). The difference in IC_50_ may indicate that ganitumab binds hybrid receptors with reduced affinity. A previously published study of ganitumab activity in the MiaPaCa2 cell line supports our present data and demonstrate that ganitumab can effectively drive tumor growth inhibition through blockade of hybrid receptor signaling [12]. In addition, published clinical pharmacokinetic data show that concentrations of ganitumab achieved on a 12 or 20 mg/kg (0.2–2 µM) every-2-week dosing schedule are well within the IC_50_ range required to inhibit hybrid receptors *in vitro* [20], [21]. These data suggest that hybrid receptor inhibition is highly probable in patients treated with clinically relevant doses of ganitumab.

Antibody-mediated receptor downregulation or receptor activation can significantly alter the activity of monoclonal antibodies against tyrosine kinase receptors [22], [23], [24]. Confocal analysis showed that IGF1R downregulation observed with some anti-IGF1R antibodies *in vitro* was due to rapid receptor internalization and enhanced degradation. Ganitumab, however, did not depend on IGF1R endosomal sequestration, since efficient IGF-1 and IGF-2 blockade was obtained *in vitro* without receptor internalization and degradation. The ability to inhibit IGF1R signaling without receptor downregulation is an important characteristic since it has been shown that internalization and downregulation of target:antibody complexes can be inefficient in some tumor cell types, depends on clathrin/caveolin endocytosis, and is rarely complete [25], [26]. Interestingly, effective inhibition of IGF1R activation by ganitumab *in vivo* was associated with partial downregulation (50% to60%) of total tumor IGF1R. This result suggests that, in human cancer models, total IGF1R downregulation may add to the ganitumab efficacy obtained by ligand inhibition. It is unclear what mechanisms are involved in triggering receptor downregulation *in vivo*. *In vitro* crosslinking studies using protein G (Amgen data on file) and prolonged *in vitro* treatments of cancer cells with ganitumab (Fig. S4) failed to reproduce the downregulation findings observed *in vivo*. The low-level activation induced by ganitumab in the Balb/C 3T3 hIGF1R and 32D hIGF1R/IRS-1 cells appeared to be specific to cells that overexpress IGF1R, since it could not be reproduced in other human cancer cell lines. In addition, it appeared to lack biological significance, since ganitumab completely inhibited proliferative responses in the presence of growth factors.

The *in vivo* properties of ganitumab were generally consistent with our *in vitro* data, but some differences were observed. The complete growth inhibition and regression of IGF1R hypersensitive tumors (32D hIGF1R/IRS-1) by ganitumab fully parallels the *in vitro* observations. The ED_50_ obtained for 32D hIGF1R/IRS-1 TGI is consistent with the value obtained with pancreatic tumor models that express both IGF1R homodimer and hybrid receptors [12]. The 5- to10-fold increase in ED_50_ relative to the EC_50_ obtained for 32D hIGF1R/IRS-1 cells *in vitro* is expected given tumor resistance to antibody penetration [27]. This ED_50_ compares well to the pharmacodynamic results obtained in a phase I clinical study of ganitumab in patients with advanced solid tumors [20]. The alignment between mouse and human data is supported by the fact that ganitumab binds and inhibits murine and human IGF1R with equal affinity and potency [28]. In contrast, the effects of ganitumab against COLO 205 and MCF-7 cells and xenografts were inconsistent and may be directly related to the dependency of these models on IGF-1 and other growth or hormonal factors for *in vivo* growth. For example, MCF-7 xenografts are dependent on estrogen, which is administered to female mice. The presence of estrogen as a driver might downplay the role of IGF-1 in this model and lead to ganitumab resistance *in vivo*. Similarly, the presence of multiple growth factors in growth media might supply COLO 205 cells with alternative pro-growth and survival signals. Access to these factors might become more limited *in vivo* leading to dependence on IGF-1 for growth and survival.

Combining IGF1R inhibitors with cytotoxic agents has been used as an approach to potentiate cellular apoptosis [29]. The results we have obtained with ganitumab and irinotecan using the COLO 205 tumor model are consistent with this therapeutic concept and show that IGF1R inhibition is compatible with cell cycle (S-phase) inhibition.

We have demonstrated that ganitumab and three new L2 domain antibodies that bind a similar epitope are distinct from antibodies that bind other IGF1R structural domains. The CR domain antibodies did not inhibit IGF-2 binding or IGF-2-mediated IGF1R activation but were more potent than L2 domain antibodies in inducing IGF1R homodimer and hybrid receptor downregulation *in vitro*. The efficacy profile of ganitumab (and L2-[A–C]) was generally equivalent to or better than the CR domain controls. However, tumor exposure was biased in favor of the CR domain antibodies due to their selectivity for human IGF1R and superior pharmacokinetic properties associated with their murine IgG1 backbones. The significance of ganitumab’s potency against IGF-2 cannot be assessed in our *in vivo* studies, since circulating IGF-2 is not detected in adult mouse models. The FnIII-1 domain antibodies were also mechanistically distinct; characterized by high agonistic activity, potent receptor downregulation, inefficient inhibition of IGF1R activation by IGF-2, and inefficient inhibition IGF1R/INSR hybrid activation by IGF-1 and IGF-2. It seems likely that receptor downregulation was primarily responsible for the inhibition of IGF-2 proliferation responses observed with the CR and FnIII-1 domain antibodies in cell-based assays. FnIII-1 antibodies were the least effective anti-IGF1R antibodies both *in vitro* and *in vivo*. These findings are consistent with the data available for antibody 1H7 [30]. However, a more recent IGF1R antibody screen has identified FnIII-1 domain-specific antibodies with low agonistic activity [31] suggesting potential mechanistic diversity within this domain.

The epitope-specific mechanisms we have described for ganitumab and other domain-specific anti-IGF1R antibodies are consistent with those obtained with murine anti-IGF1R monoclonal antibodies [13], [14], [15], [30], [32]. Our study suggests that in addition to the structural and pharmacokinetic distinctions that exist among the IGF1R antibodies in clinical development, mechanistic differences with important biological consequences may exist. Understanding these differences will be required to ensure optimal clinical development of this class of anti-cancer therapeutics.

## Supporting Information

Figure S1 **Locations of the binding epitopes on IGF1R for each monoclonal antibody.**
(TIF)

Figure S2 **The effect of IGF1R domain-specific antibodies on INSR activation.** Serum-starved CHO cells engineered to overexpress the INSR-B isoform were treated with increasing concentrations of representative anti-IGF1R antibodies. (A). Ganitumab; (B). Mab 391; (C). F1-B; (D). control antibody in the presence and absence of growth factors (16 nM IGF-1, 32 nM IGF-2, 4 nM INS). The murine anti-INSR antibody 47-9 was used as a positive control.
(TIF)

Figure S3 **The effect of IGF1R domain-specific antibodies on COLO 205 and MCF-7 growth.** The confluence of cells cultured with 1 µM control anti-CD20 antibody or the indicated anti-IGF1R antibody in 96-well format was continuously monitored with phase contrast microscopy (IncuCyte™). Antibody was added at the time of cell plating. (A–C). COLO 205 cells (15,000 per well) were plated (in duplicate) in RPMI plus 10% FBS. (D–F). MCF-7 cells (10,000 per well) were plated (5 replicates) in RPMI plus 10% FBS. Straight lines were generated to the linear regions of log-transformed data using a nonlinear subroutine (GraphPad Prism).
(TIF)

Figure S4 **Characterization of IGF1R and INSR internalization and degradation in MCF-7 breast cancer cells.** A. MCF-7 cells in DMEM (high glucose) plus 10% FBS were treated with 250 nM of ganitumab, Mab 391, or F1-B over a 2-week period to determine their long-term effects on IGF1R expression. The antibody was replenished when the cells were subcultured. All signals were normalized to the IGF1R signal obtained with the control antibody at each time point. B. Mice with established (200–300 mm^3^) subcutaneous MCF-7 tumors were treated with ganitumab, Mab 391, or F1-B (300 µg/dose, IP, twice weekly). At the indicated time points, three animals were sacrificed, and IGF1R levels were determined. The % control is the signal obtained for an individual animal divided by the mean for the control antibody multiplied by 100 for each treatment group. Total INSR level was determined in the same cell extracts (C) and tumor extracts (D) used for the long-term analysis of IGF1R.
(TIF)

Figure S5 **Antibody effects on IGF1R and INSR activation by IGF-1 and IGF-2 in MCF-7 cells.** Determination of antibody IC_50_ for IGF1R (A–C) or INSR (D–F) inhibition. Serum-starved MCF-7 cells were treated for 20 minutes simultaneously with either IGF-1 (2 nM) or IGF-2 (8 nM) and antibody as indicated. Total (t) and phosphorylated (p) IGF1R were determined (in duplicate) after αIR3 and Mab 391 treatment using an MSD assay with F1-B as the capture agent.
(TIF)

Table S1 **Effects of IGF-1, IGF-2, and INS on IGF1R and INSR Activation.**
(DOCX)
