# Supplementary figures and images for: Epitope-Specific Mechanisms of IGF1R Inhibition by Ganitumab
Source: PLoS One. 2013 Feb 1;8(2):e55135. doi: 10.1371/journal.pone.0055135 (PMC3562316; doi:10.1371/journal.pone.0055135)

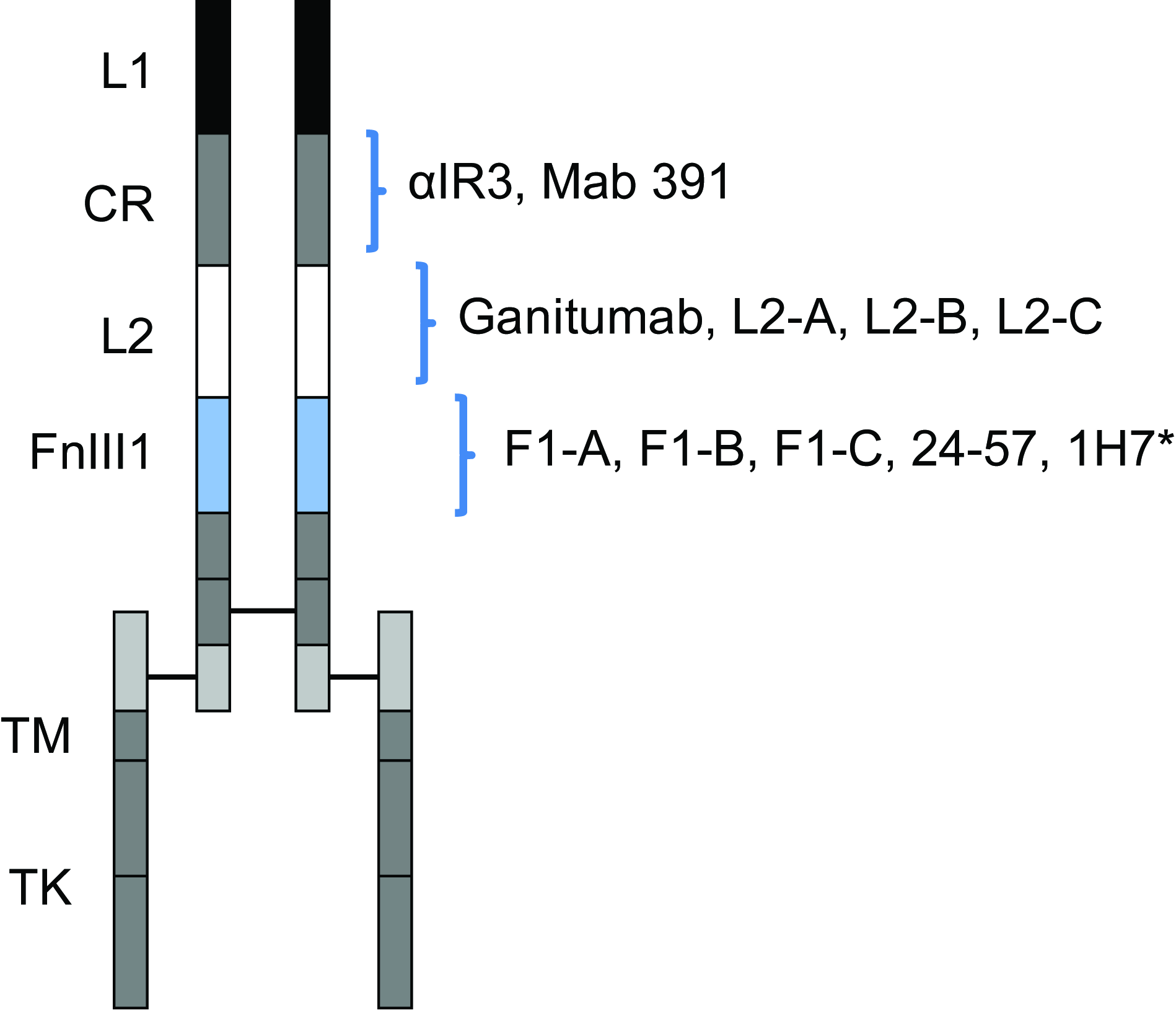

Supplement: Figure S1 — Locations of the binding epitopes on IGF1R for each monoclonal antibody. (TIF) [file pone.0055135.s001.tif]

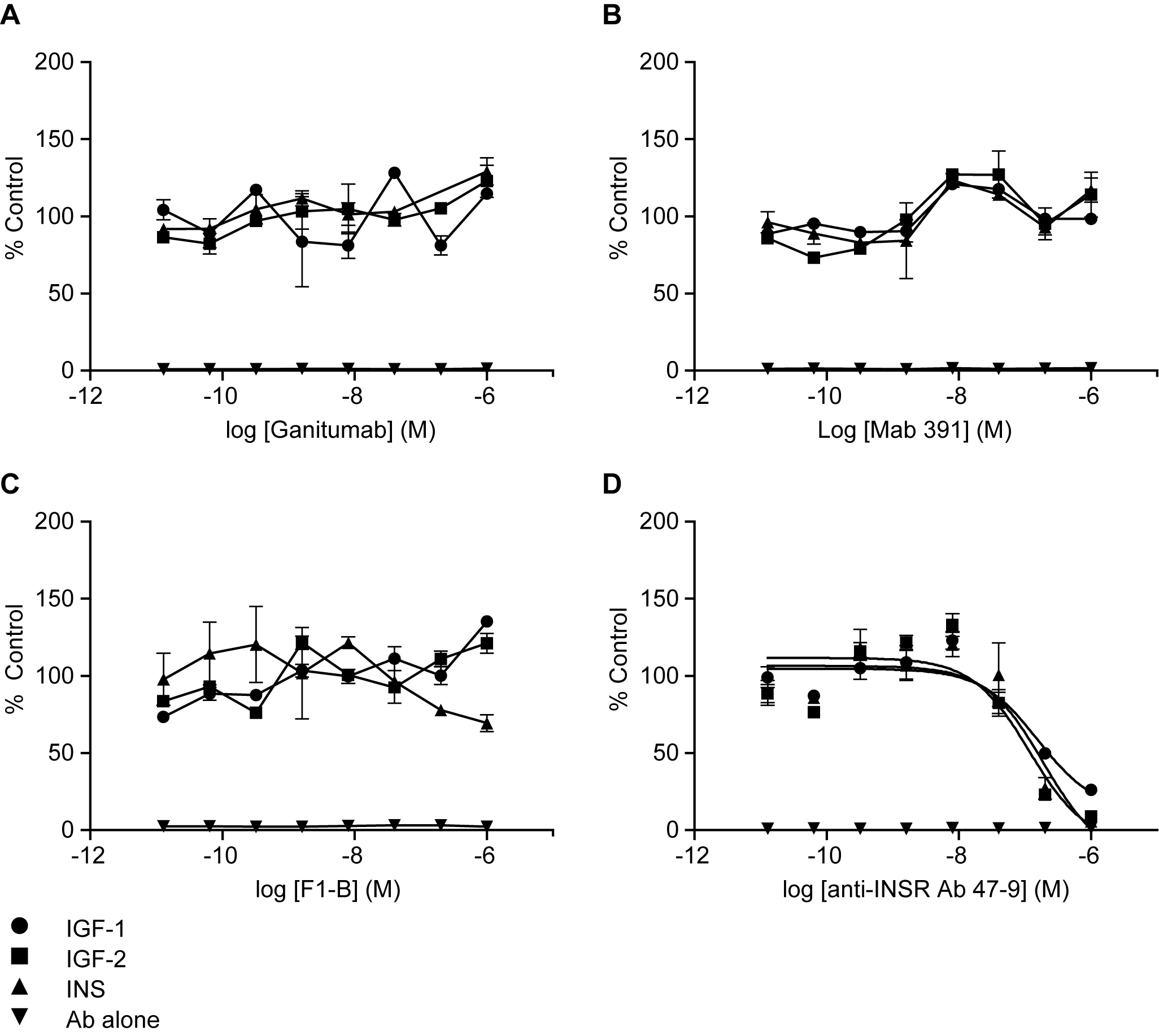

Supplement: Figure S2 — The effect of IGF1R domain-specific antibodies on INSR activation. Serum-starved CHO cells engineered to overexpress the INSR-B isoform were treated with increasing concentrations of representative anti-IGF1R antibodies. (A). Ganitumab; (B). Mab 391; (C). F1-B; (D). control antibody in the presence and absence of growth factors (16 nM IGF-1, 32 nM IGF-2, 4 nM INS). The murine anti-INSR antibody 47-9 was used as a positive control. (TIF) [file pone.0055135.s002.tif]

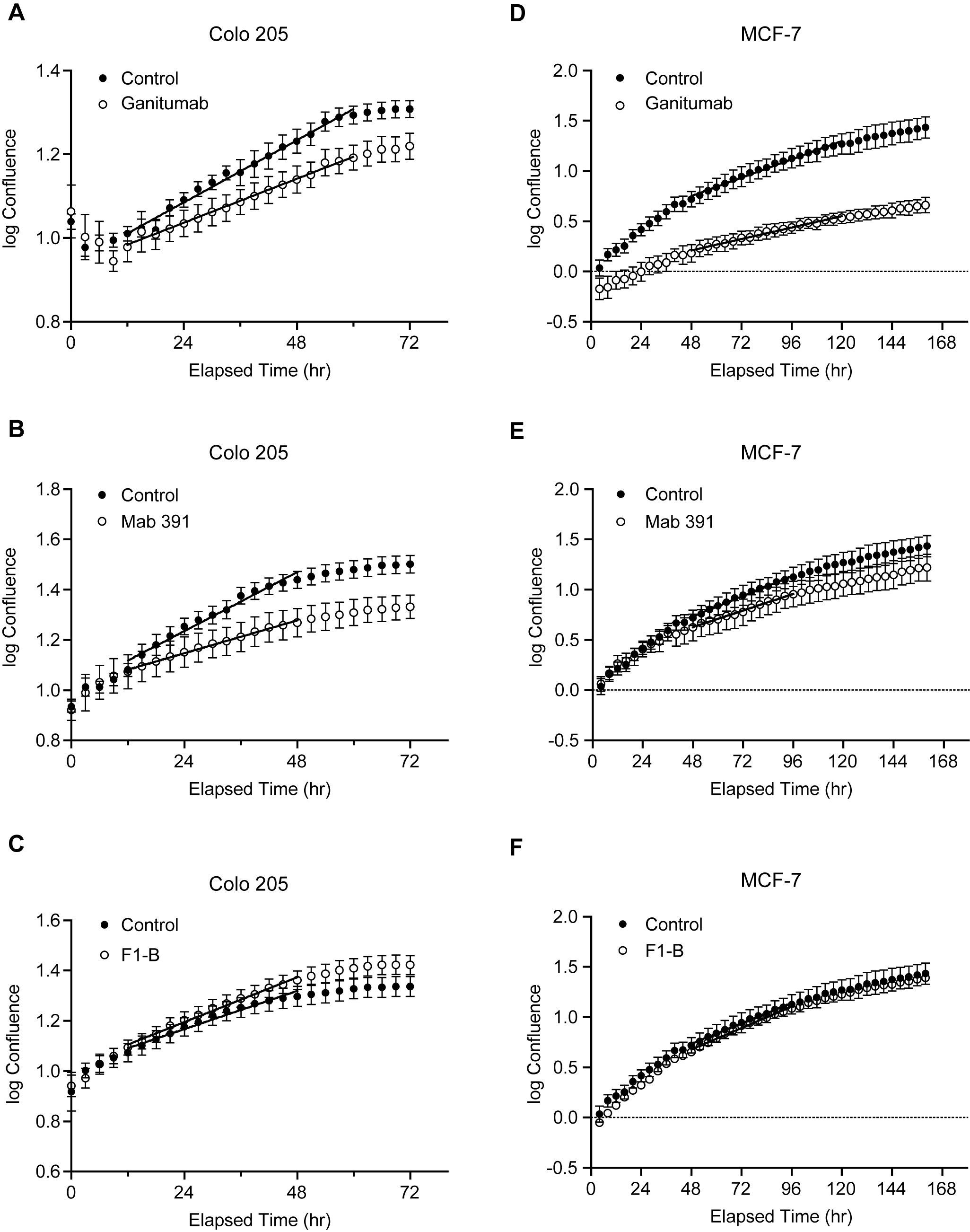

Supplement: Figure S3 — The effect of IGF1R domain-specific antibodies on COLO 205 and MCF-7 growth. The confluence of cells cultured with 1 µM control anti-CD20 antibody or the indicated anti-IGF1R antibody in 96-well format was continuously monitored with phase contrast microscopy (IncuCyte™). Antibody was added at the time of cell plating. (A–C). COLO 205 cells (15,000 per well) were plated (in duplicate) in RPMI plus 10% FBS. (D–F). MCF-7 cells (10,000 per well) were plated (5 replicates) in RPMI plus 10% FBS. Straight lines were generated to the linear regions of log-transformed data using a nonlinear subroutine (GraphPad Prism). (TIF) [file pone.0055135.s003.tif]

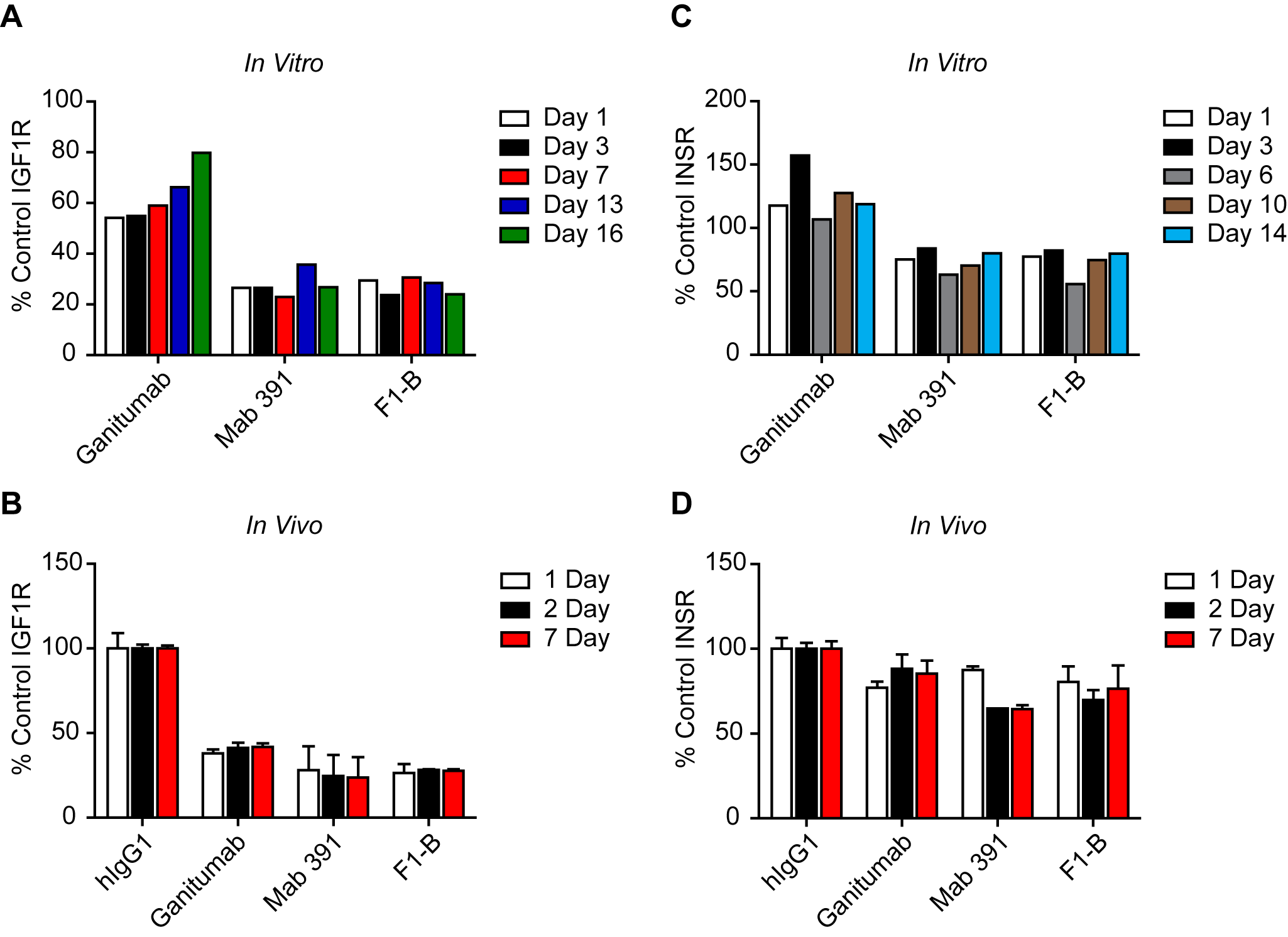

Supplement: Figure S4 — Characterization of IGF1R and INSR internalization and degradation in MCF-7 breast cancer cells. A. MCF-7 cells in DMEM (high glucose) plus 10% FBS were treated with 250 nM of ganitumab, Mab 391, or F1-B over a 2-week period to determine their long-term effects on IGF1R expression. The antibody was replenished when the cells were subcultured. All signals were normalized to the IGF1R signal obtained with the control antibody at each time point. B. Mice with established (200–300 mm3) subcutaneous MCF-7 tumors were treated with ganitumab, Mab 391, or F1-B (300 µg/dose, IP, twice weekly). At the indicated time points, three animals were sacrificed, and IGF1R levels were determined. The % control is the signal obtained for an individual animal divided by the mean for the control antibody multiplied by 100 for each treatment group. Total INSR level was determined in the same cell extracts (C) and tumor extracts (D) used for the long-term analysis of IGF1R. (TIF) [file pone.0055135.s004.tif]

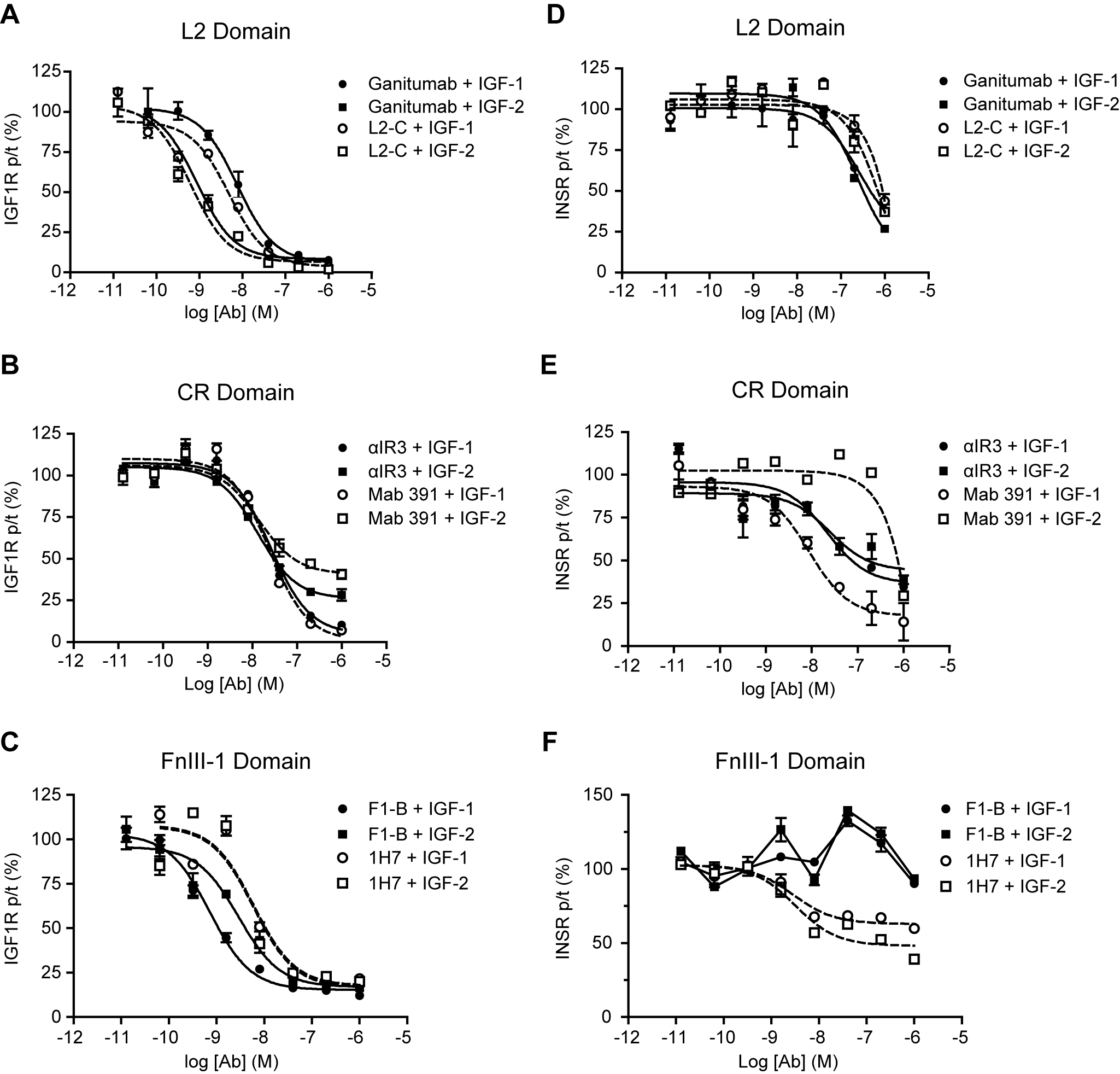

Supplement: Figure S5 — Antibody effects on IGF1R and INSR activation by IGF-1 and IGF-2 in MCF-7 cells. Determination of antibody IC50 for IGF1R (A–C) or INSR (D–F) inhibition. Serum-starved MCF-7 cells were treated for 20 minutes simultaneously with either IGF-1 (2 nM) or IGF-2 (8 nM) and antibody as indicated. Total (t) and phosphorylated (p) IGF1R were determined (in duplicate) after αIR3 and Mab 391 treatment using an MSD assay with F1-B as the capture agent. (TIF) [file pone.0055135.s005.tif]
