# Supplementary material for: Epitope-Specific Mechanisms of IGF1R Inhibition by Ganitumab
Source: PLoS One. 2013 Feb 1;8(2):e55135. doi: 10.1371/journal.pone.0055135 (PMC3562316; doi:10.1371/journal.pone.0055135)
Supplement: Table S1 — Effects of IGF-1, IGF-2, and INS on IGF1R and INSR Activation. (DOCX) [file pone.0055135.s006.docx]

## **Table S1. Effects of IGF-1, IGF-2, and INS on IGF1R and INSR Activation.**

| **Cell Line** | **Receptor** | **Mean No. of Receptors Per Cell** | **IGF-1** | | **IGF-2** | | **INS** | |
| --- | --- | --- | --- | --- | --- | --- | --- | --- |
|  |  |  | **EC_50_ (nM)** | **MI** | **EC_50_ (nM)** | **MI** | **EC_50_ (nM)** | **MI** |
| 32D hIGF1R/IRS-1 | IGF1R | 55,000 | 0.5 (0.15–1.5) | 62 | 9.3 (7.1–12) | 134 | ND | ND |
|  | INSR | NA | NA | ND | NA | ND | ND | ND |
| Balb/C 3T3 hIGF1R | IGF1R | 500,000 | 2.7 (1.0–7.4) | 30 | 33 (26–44) | 52 | ND | ND |
|  | INSR | NA | NA | ND | NA | ND | ND | ND |
| COLO 205 | IGF1R | 22,000 | 0.2 (0.1–0.4) | 32 | 2.1 (1.4–3.1) | 43 | ND | ND |
|  | INSR | 7,000 | 0.4 (0.3–0.6) | 36 | 7.0 (2.1–23) | 41 | 8.6 (3.2–24) | 17.5 |
| MCF-7 | IGF1R | 88,000 | 0.2 (0.05–0.5) | 54 | 3.3 (2.0–5.5) | 117 | ND | ND |
|  | INSR | 8,000 | 0.4 (0.2–0.7) | 32 | 9.9 (3.2–30) | 42 | 3.0 (1.0–8.9) | 24 |

Quantitative flow cytometry was used to determine the mean number of IGF1R and INSR per cell. Growth factor titrations were performed as described previously and the EC_50_s for receptor activation were determined as described previously [[12](#_ENREF_12)]. The 95% confidence intervals are given in parentheses.

Abbreviations: MI: maximum induction (fold over baseline); ND: not done (EC_50_ measurements were not determined because saturation was not achieved at 200 nM ligand concentration); NA: not applicable (the assays did not detect murine IGF1R or murine INSR).
